# The Effectiveness of Ultrasound Biomicroscopic and Anterior Segment Optical Coherence Tomography in the Assessment of Anterior Segment Tumors: Long-Term Follow-Up

**DOI:** 10.1155/2020/9053737

**Published:** 2020-06-16

**Authors:** Joanna Konopińska, Łukasz Lisowski, Ewa Wasiluk, Zofia Mariak, Iwona Obuchowska

**Affiliations:** Department of Ophthalmology, Medical University of Białystok, M. Sklodowska-Curie 24A STR, 15-276 Białystok, Poland

## Abstract

**Background:**

Differential diagnosis and follow-up of small anterior segment tumors constitute a particular challenge because they determine further treatment procedures. The aim of this study was to evaluate the efficacy of the UBM (ultrasound biomicroscopy) and AS-OCT (anterior segment optical coherent tomography) in distinguishing different types of anterior segment lesions.

**Methods:**

It was a retrospective, noncomparative study of case series of 89 patients with the suspicion of anterior segment tumor referred to the Ophthalmology Clinic, Medical University of Białystok, Poland, between 2016 and 2020. UBM was used to assess tumor morphology including height, location, and internal and external features. In cases in which UBM did not provide enough data, the AS-OCT images were analyzed. The data on demographics, best corrected visual acuity (BCVA), intraocular pressure (IOP), and rate of complications were also collected. Patients were followed up from 1 to 48 months.

**Results:**

The mean observation period was 26.61 ± 16.13 months. Among the patients, there were 62 women and 27 men at a mean age of 55.59 ± 19.48 (range: from 20 to 89 years.) The types of tumors were cysts (41%), solid iris tumors (37.1%), ciliary body tumors (7.9%), peripheral anterior synechiae (PAS 3.4%), corneal tumors (4.5%), and others (5.6%). Patients with cysts were younger than patients with solid iris tumor (*p*=0.002). Women had a cyst as well as solid iris tumor more frequently than men, but less often a ciliary body tumor (*p* < 0.05). The horizontal size of tumor was positively correlated with patients' age (*r*_*s*_ = 0.38 and *p*=0.003) and negatively correlated with visual acuity (*r*_*s*_ = −0.42 and *p*=0.014). During the 4 years of diagnosis, only 2.2% of lesions exhibited growth (growth rate of 0.02 mm per year). Among 15 cases in which visualization with UBM was not satisfactory (mostly iris nevi), AS-OCT was helpful in diagnosis of 13 patients.

**Conclusions:**

Both UBM and AS-OCT are effective methods in detection and diagnosis of tumors of the anterior eye segment, but in some cases, AS-OCT adds additional value to the diagnosis. Many lesions can be managed conservatively because they did not demonstrate growth during 4 years of the follow-up period.

## 1. Introduction

Detection and monitoring of anterior segment tumors is a major challenge due to their location, which makes direct visualization of these lesions in a basic ophthalmological examination difficult. Consequently, many tumors remain undiagnosed for a long time or are diagnosed too late when they are large enough to produce ocular symptoms. Therefore, the use of additional tests for the early diagnosis of anterior segment tumors is necessary. These examinations should enable the assessment of tumor parameters such as size, location, infiltration of surrounding structures, and growth rate. This is now possible due to the development of such techniques of imaging the anterior segment of the eye as high-frequency ultrasound biomicroscopy (UBM) and anterior segment optical coherence tomography (AS-OCT).

UBM is recognized as the gold standard in the imaging of anterior segment tumors [1]. This test uses high-frequency ultrasound, from 20 MHz to 100 MHz, which allows a resolution of 20–50 *μ*m, with tissue penetration up to 4–7 mm. With its help, in a noninvasive and detailed way, it is possible to visualize the anatomy of the anterior segment of the eye, especially structures inaccessible to visualization in a standard examination using a slit lamp. These include, for example, the anterior chamber angle, ciliary body, the peripheral part of the lens, haptens of artificial intraocular lens (IOL), or even the outermost parts of the retina. UBM provides also accurate biometric measurements of assessed eyeball structures [1–4].

Modern AS-OCT devices use a light beam with a wavelength of 1310 nm, which allows us to obtain high axial resolution, even up to 5–7 *μ*m with the spectral-domain OCT. However, AS-OCT limitation includes a penetration depth of 3–6 mm at a scan width up to 6–16 mm and poor penetration through the iris pigment epithelium, which in some cases of lesions located behind the iris allows only visualization of their anterior walls. It is a noncontact and quick test, and it is a perfect complement to UBM [4, 5].

Although several studies comparing AS-OCT with UBM in assessment of anterior segment tumors [5–8] have been published, there is very little information on the long-term follow-up of these tumors in the literature.

The purpose of this study is to evaluate the characteristics of anterior segment tumors, which were referred to the Ophthalmology Clinic Medical University of Bialystok between 2016 and 2020, with the usage of these two methods of imaging, i.e., UBM and AS-OCT. We tried to determine which techniques provide better visualization and characterization of certain anterior segment tumors. We have also reported our experiences with long-term follow-up of these tumors to detect the growth and the rate of other morphological features related to the higher risk of malignancy.

## 2. Materials and Methods

This study was approved by the Bioethics Committee of the Medical University of Białystok in accordance with the ethical standards of the 1964 Declaration of Helsinki and its later amendments or comparable ethical standards. All the patients gave written, fully informed consent for the examination and the use of their clinical data for publication.

We conducted a retrospective review of the medical records and electronic images of all patients with suspected anterior segment tumors who were examined at the Department of Ophthalmology, Medical University in Bialystok between April 2016 and February 2020. We obtained the following data from medical records: gender, age, BCVA, IOP, anterior segment clinical evaluation, images obtained with UBM (Aviso S, Quantel Medical, Paris, France v 5.0.0), and AS-OCT (Spectralis Tracking Laser Tomography, Heidelberg Engineering).

UBM was performed in all patients, and this test was considered the gold standard in the diagnosis of anterior segment tumors [1]. UBM was performed by two experienced researchers (JK and ŁL) according to the method described earlier [8] with a 50 MHz transducer. Images were obtained at the radical meridian through the largest tumor thickness using an eyecup filled with 1% methylcellulose and distilled water.

Ultrasound images were evaluated for the type of lesion, size, location, penetration into the anterior chamber or outside the iris pigment epithelium, echogenicity, external structure (regular/irregular), infiltration of surrounding structures, iris pigmentation, and documented growth. The dimensions of the iris tumors were determined as the largest dimension of the base and the largest dimension of the height, drawn in a line perpendicular to each other, with an accurate determination of the o'clock position. If the lesion was in the cornea, its thickness was not included in the measurement of the size of the lesion (as long as the resolution of the test allowed to distinguish this boundary).

The height of the ciliary body tumors was measured perpendicularly from the internal surface of the sclera to the tumor surface at the thickest portion of the tumor. The growth of a lesion was defined as an increase of its height by at least 20% in comparison with the previous measurement in two separate tests [9]. Imaging parameters were set uniformly during all tests: using a gain of 100 decibels (db), Dyn = 50 db and Tgc = 0 db, and a time-gain control of 5 db/min.

In cases where no change was seen in the UBM image, the patient underwent AS-OCT. This test was performed by an experienced researcher (ŁL) using the IR20°ART + OCT 15° (3 mm) protocol, and the anterior chamber evaluation module was always used in the same way. To minimize the risk of distortion, it was ensured that the light beam ran perpendicularly to the iris and the tested lesion, and corneal reflex was clearly visible. The best quality scan was used for the analysis.

Based on ultrasound assessment, the lesions were classified into the following groups: cysts, solid iris lesions, ciliary body tumors, peripheral anterior synechiae (PAS), corneal tumors, and others. More than 3 cysts in the eye were classified as multiple cysts [10]. Follow-up visits were scheduled at six-month intervals. If disturbing symptoms (an increase in IOP; presence of tortuous and dilated vessels going towards the lesion) were observed, the frequency of visits was higher and adapted to the local condition.

### 2.1. Statistical Analysis

Statistical analysis was performed using R 3.5.1. The studied variables were presented with the use of descriptive statistics. Nominal variables were compared between groups by Fisher's exact test. The normality of the distribution of quantitative variables was assessed using the Shapiro–Wilk test, skewness and kurtosis indicators, and visual assessment of histograms. Group comparisons for quantitative data were performed by the Mann–Whitney *U* test or the Kruskal–Wallis test with the Dunn test, when appropriate. The Bonferroni correction was employed because of multiple comparisons. A comparative analysis of the tumor size with individual tests was performed with the Wilcoxon test for dependent measurements. Correlation of the tumor size with selected quantitative parameters was checked by Spearman's rank correlation coefficient. The significance level *α* = 0.05 was used, and all statistical tests were two-sided.

## 3. Results

The study involved 89 patients with suspected anterior segment tumor. They were 62 women and 27 men at an average age of 55.59 ± 19.48 years, with a range of 20–89 years.

**Figure 1:**
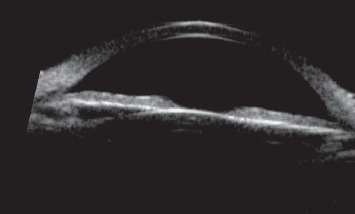
A patient with iris nevus which was not visualized in UBM.

**Figure 2:**
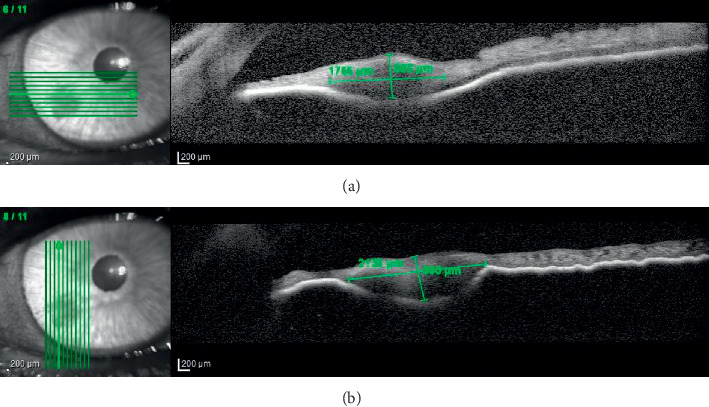
Well-visible iris nevus on the AS-OCT image in the same patient.

**Figure 3:**
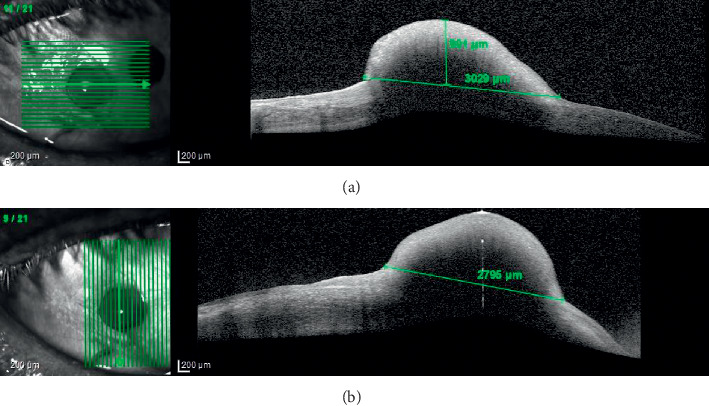
A well-visible boundary of corneal leukoma.

Tumor-like lesions were revealed in UBM in 74 people (83% of the group). In 13 (14.6%) subsequent cases, the diagnosis was confirmed by AS-OCT. Only in two patients with iris nevi, visible in the slit lamp, it was not possible to visualize the change in either UBM or AS-OCT. Finally, it was found that cysts (*n* = 37, 42%) and solid iris lesions (*n* = 33, 37%) were the most common anterior segment lesions in the study group. Other less-frequent lesions were ciliary body tumors (*n* = 7, 7.9%), corneal tumors (*n* = 4, 4.5%), PAS (*n* = 3, 3.4%), and other lesions (*n* = 5, 5.6%). Other lesions included 2 cases of corneal leukoma, conjunctival nevus, thinning of the sclera with a translucent choroid after childhood esophoria surgery, and IOL decentration causing iris elevation. UBM provided effective visualization in 74 cases (80.1%). However, in 15 cases, UBM did not show tumor mass, and these were 7 solid iris lesions (Figure 1.), 3 PAS cases, 1 IOL displacement, 1 conjunctival nevus, 2 cases of corneal leukoma, and 1 case of scleral thinning after childhood esophoria surgery. The AS-OCT images of these patients were analyzed. In 5 cases, the lesion was revealed, namely, iris nevus (Figure 2). In 2 cases of corneal leukoma, AS-OCT could accurately determine the boundary between the cornea and the growing lesion (Figure 3). In the other 2 cases, the lesion could not be visualized either.

Tumor size measurements were made based on UBM. The average values of the base width and height of all measured tumors are presented in Table 1.

In addition, the mean horizontal and vertical dimensions of the solid iris tumor were significantly smaller than those of the ciliary body tumor *p*=0.018 and *p* < 0.001, respectively, and the horizontal dimension of the cyst was also significantly smaller from that of the corneal tumor (*p*=0.017).

A statistically significant difference was found for both horizontal and vertical tumor dimensions depending on the type of lesion (Table 3). The mean horizontal and vertical dimensions of the cyst were significantly different than the ciliary body tumor dimension (*p* < 0.001 and *p*=0.017, respectively). In addition, the mean horizontal and vertical dimensions of solid iris tumor were significantly different than those of ciliary body tumor (*p*=0.018 and *p* < 0.001, respectively). The mean horizontal cyst dimension was also significantly different from that of the corneal tumor (*p*=0.017).

The mean age of the patients was significantly statistically different (*p*=0.006) between the patients with particular types of tumor (Table 4). A post hoc analysis indicated that patients with cysts were much younger than patients with solid iris tumor (*p*=0.002). A significant relationship between tumor type and gender was also found. Women had a cyst more frequently than men (45% of women and 33% of men) as well as solid iris tumor (36% vs. 26%, respectively). In turn, men had a ciliary body tumor (15% of men and 5% of women) and other changes (15% vs. 2%, respectively) more frequently than women (Table 5).

Comparison of BCVA and IOP values depending on the type of tumor revealed that patients with cysts had significantly higher BCVA than patients with other lesions (Table 6). However, no correlation was found between the IOP value and tumor type (Table 7).

A tumor horizontal size was positively correlated with patients' age (*r*_*s*_ = 0.38, *p*=0.003) and negatively correlated with visual acuity (*r*_*s*_ = −0.42, *p*=0.014). Both the demonstrated correlations had a moderate strength. The relationship between the horizontal and vertical dimensions of the tumor and the IOP value was not confirmed (Table 8).

The assessment of the anterior segment of the eye in the slit lamp revealed additional symptoms besides the tumor in 5 patients. In 2 cases of ciliary body tumor, the following complications were observed: 1 sectoral cataract and 1 inflammatory reaction in the anterior uvea. Increased IOP values were found in 3 patients with multiple cysts. These patients were treated with the Nd: YAG laser to perforate the cyst walls and drain the internal fluid according to the earlier described technique [11]. After the procedure, normalization of IOP was observed in two of these patients; in one of them, it was necessary to include hypotensive treatment.

Follow-up examinations were routinely performed on all patients every 6 months, with the exception of 10 individuals who already had disturbing symptoms during the first examination that could indicate malignancy. These were as follows: all cases of ciliary body tumors (7 patients), 1 case of iris tumor due to visible additional symptoms: tortuous vessels going from the angle of infiltration to the tumor mass, 1 case of iris tumor and concomitant sectoral cataract, and 1 case of iris tumor with signs of infiltration into the filtration angle. These patients were immediately referred for further diagnostics and possible treatment to a specialist center of intraocular cancer treatment.

In 2 patients, tumor growth by ≥ 20%, when compared to the first examination, was confirmed by a follow-up. These patients were immediately referred for further diagnosis, like in the abovementioned cases. Of all the patients referred to another ophthalmology center, 2 returned with confirmation of the malignant process. They underwent brachytherapy and were referred to further observation at the place of residence. In 3 patients, the tumor process was excluded, and further follow-up was recommended. The fate of the remaining patients is unknown to us. Ultimately, in the remaining patients, the follow-up ranged from 1 to 48 months. The average follow-up length was 26.61 ± 16.13 months.

## 4. Discussion

Iris elevation or focal discoloration in the anterior segment of the eye is always an alarming symptom for the ophthalmologist. In our study, it turned out that in 92% of cases, this translated into the presence of a tumor (42% of cysts, 37% of solid iris tumor, 7.9% of ciliary body tumor, or 4.5% of corneal tumor), and only in 8% of cases, the cause may be different (PAS, scleral thinning, IOL decentration, or corneal leukoma).

Documenting objective tumor growth is always a challenge, and without the use of additional imaging tools, it cannot be precise. Taking a photograph of the anterior segment of the eye may be helpful but only allows imaging of the lesion surface. Sequential UBM allows detection of the tumor size change. Therefore, it allows, in some cases, to avoid invasive diagnostics, i.e., fine-needle aspiration or iridocyclectomy [4, 11]. In our study, tumor growth was observed only in 2.2% of patients. Other features (i.e., tumor size, presence of abnormal tortuous vessels, sectoral cataract, and inflammation in the anterior chamber) resulted in the referral for further oncological diagnosis of 10 patients (11%). In the study by Shields et al., in 200 cases, 24% were finally qualified as lesions requiring further oncological diagnosis [12].

Cysts (42%) were the most common change in our study group. Cysts in the anterior segment of the eye can be classified as primary or secondary ones. Primary cysts are epithelial, while secondary ones may be the result of implantation, tumor metastasis, parasitic infections, or chronic use of miotics. Primary cysts rarely cause complications or impair BCVA [4]. They have thin, regular walls and a hypoechogenic interior. Secondary cysts involve the risk of many complications such as corneal edema, uveitis, secondary angle-closure glaucoma, astigmatism, or cataracts due to lens compression. These disorders usually involve significant visual impairment [1, 11]. In our study, in three cases of multiple and binocular cysts, we observed an increase in IOP, but we did not observe cases with reduced BCVA. Implantation cysts originating from the conjunctival epithelium, cornea, or eyelid skin are the results of penetrating trauma or surgical intervention. They can take the form of compact masses (pearl-like cysts), reservoirs filled with liquid, or they can cause endothelial hyperplasia. They are usually large (about 5 mm in cross section) and have thick walls (about 0.4 mm). They may contain serous, echo-negative fluid content (serous cysts). It is very important to distinguish the cyst from the echo-negative space inside the tumor that corresponds to the focus of necrosis or the lumen of a large blood vessel.

However, UBM does not allow to distinguish serous content, erythrocytes, or inflammatory cells, so histopathology still plays a key role in such cases [1]. Their growth varies; initially, they can grow rapidly and later remain unchanged. By reaching large sizes, they can overgrow the iris, causing its atrophy, as well as they penetrate into the posterior chamber. In our study, there were 5 secondary cysts: 1 caused by trauma in childhood, 2 previous surgeries: phacotrabeculectomy and ECCE, and in 2 cases, the reason was not revealed.

The use of AS-OCT is of limited significance in the case of central cysts, under the iris pigment epithelium. Numerous studies confirm UBM advantage over AS-OCT in detecting these changes [3, 13–15]. The pigment epithelium absorbs light to a large extent, and its cells are linked tightly by means of desmosomes, as a result of which it is impossible to visualize the circumference of the cyst. Peripheral cysts, located in the iridociliary sulcus, are partially covered with colorless epithelium, and the links between its cells are less tight and have gaps, so their visualization with AS-OCT is possible at least partially [1].

There are studies describing the family occurrence of iris cysts with dominant autosomal inheritance [16, 17]. In these cases, multiple cysts often cover more than 180° of the filtration angle. In our study group, we had 1 case of siblings (brother and sister) with multiple binocular cysts. In such cases, it would be worth extending the diagnostics to other family members. Centrally located primary cysts in adults are usually asymptomatic, and even signs of spontaneous regression have been observed, although they may also slowly increase with time [18]. No such cases were observed in our study. Sometimes, cysts can cause an increase in IOP due to the obstruction of filtration angle or clogging of the openings of trabecular meshwork by mucus released from the secondary cyst [18]. In our study, only 3 patients had an increase in IOP, and these were multiple cysts that covered >180° of the filtration angle. Our study confirmed the conclusions of Shields et al. that primary iris cysts rarely progress and affect BCVA and IOP levels [12]. In Shield's study, they accounted for 21% of cases in the group of patients referred for examination with suspected tumor. Binocular and multiple cysts accounted for 37.8% [10] in another study and 16% (6 cases) in our study.

The second most common diagnosis among our group was solid iris tumors. They occur in the form of localized foci of pigmentation of the iris, which are flat or slightly elevated. Sometimes, these lesions can grow and infiltrate surrounding tissues [19]. The diagnosis of this type of lesions is particularly important, especially when they reveal signs of pupil displacement, ectropion uvea, or cataracts in the adjacent quadrant, due to the possibility of melanoma on their basis.

Typically, the iris tumors look like weak-reflective plaques surrounding the thickened iris stroma. A lesion close to the base of the iris can cause its deflection [10]. Certain characteristics of neoplastic transformation, i.e., location, presence of abnormal vessels, or uneven edge of the lesion, can be assessed during the slit-lamp examination. However, imaging of penetration through the pigment lamina, confirmation of the growth of the lesion, infiltration of structures, or confirmation of uneven echogenicity are not possible without the use of additional devices [19]. In our study, this was confirmed in 4 cases of iris tumors (12%).

In 7 patients with iris tumor, the ultrasound image could not be obtained due to the lack of reflections caused by the resolution of the test. These were mainly the cases of iris nevi. Of these, in 5 patients, AS-OCT showed high-resolution images on the basis of which it could be concluded that the nevus does not penetrate through the iris pigment epithelium, which is an important prognostic feature. AS-OCT may also be a useful alternative in imaging small, nonpigmented iris tumors (with a thickness of not more than 1.3 mm and a base width of not more than 3 mm) [20].

In the study by Hau et al. [13], it was shown that the possibility of accurate imaging of iris nevi with dimensions ≤2 mm of the base width and 0.6 mm in height was 87.1% of all cases, and in the study by Razzaq et al., it was as much as 96% [21]. Moreover, greater precision in determining the tumor size that is possible with AS-OCT (no additional echoes as in the case of UBM) allows the calculation of an adequate brachytherapy dose for confirmation of melanoma [21]. However, if the lesion was larger or penetrated into the area behind the iris, it was not possible to visualize its entire volume. In this case, as well as in highly pigmented lesions, ultrasound imaging is more helpful due to better penetration compared to light energy.

All cases of ciliary body tumors were referred for further oncological diagnostics, since there are studies showing that melanomas of this area are more aggressive than melanomas of the iris or choroid due to the rich vascularization of the ciliary body, which increases the risk of distributing cancer cells with blood or large initial tumor size related to its late detection [9]. In addition, at the time of diagnosis, they were large, on average 5.72 × 4.75 mm. Moreover, in each of these cases, there was a reduced BCVA and uveitis in one case.

There are several weak spots in our study. It was a retrospective study, and tumor growth criteria were retrospectively defined. Moreover, UBM is characterized by intraobserver and interobserver variability depending on the experience of the ultrasound technician [22–24]. Measurement of the greatest thickness in lesions with irregular contours can also be difficult, although it is much easier to determine the exact position of the transducer during the measurement. Despite these drawbacks, a large study group and long observation period can constitute the advantage of the study.

## 5. Conclusions

In conclusion, in the diagnostics of cysts and small anterior ocular tumors, UBM provides key information about their exact location and anatomical structure, i.e., echogenicity of the inside of the lesion, its structure, shape, contour (regular, irregular), wall thickness, and location relative to the surrounding structures (infiltration) and an increase in the size of the cyst or tumor visible in subsequent tests. These are important diagnostic and prognostic parameters. In some cases, when the lesion cannot be visualized by ultrasound, AS-OCT is helpful in diagnosing them and taking further therapeutic steps due to the possibility of obtaining a high-resolution image. UBM is still the gold standard in the diagnosis of anterior segment tumors, and AS-OCT is a valuable complement. Long-term observation of the lesions shows that most of the lesions are mild and asymptomatic, and they do not cause complications and do not require treatment. However, it should be remembered that histopathology is still of key importance for diagnosis and implementation of appropriate treatment for anterior segment tumors.

## Figures and Tables

**Table 1:** Tumor size mean values, median values, standard deviations, and the range at the first visit.

Tumor size (mm)	*n*	Mean	SD	Median	*Q1*–*Q3*	Range
Base width	74	2.97	2.32	2.36	1.79–2.90	0.96–12.87
Height	74	1.38	0.87	1.10	0.81–1.41	0.48–4.60

Comparison of average tumor sizes does not indicate significant statistical differences between men and women (Table 2).

**Table 2:** Tumor size mean values, median values, standard deviations, and the range by gender.

Gender	Base width (mm)				Height (mm)			
	*n*	Mean (SD)	Median (range)	*p*	*n*	Mean (SD)	Median (range)	*p* ^*∗*^
Females	53	2.95 ± 2.34	2.29 (0.96; 12.87)	0.512	52	1.40 ± 0.90	1.09 (0.48; 4.60)	0.886
Males	21	3.03 ± 2.31	2.41 (1.31; 11.33)		19	1.33 ± 0.80	1.21 (0.53; 3.93)	

^*∗*^Mann–Whitney *U* test.

**Table 3:** Tumor size mean values, median values, standard deviations, and the range by tumor types.

Tumor type	Base width (mm)				Height (mm)			
	*n*	Mean (SD)	Median (range)	*p*	*n*	Mean (SD)	Median (range)	*p* ^*∗*^
Cyst	37	2.07 ± 0.91	1.87 (1.04; 5.64)^***a,b***^	<0.001	37	1.20 ± 0.60	1.09 (0.63; 4.04)^***d***^	<0.001
Solid iris tumor	26	2.40 ± 0.70	2.29 (0.96; 3.83)^***c***^		26	0.92 ± 0.37	0.81 (0.48; 2.15)^***e,f***^	
Ciliary body tumor	7	5.72 ± 3.02	4.75 (2.41; 11.33)^***a,c***^		7	2.65 ± 1.06	3.18 (1.14; 3.93)^***d,f***^	
Corneal tumor	4	6.34 ± 4.28	6.23 (2.50; 10.38)^***b***^		4	1.77 ± 0.89	1.72 (0.86; 2.79)	

^*∗*^Kruskal–Wallis test; *a–f*: significant differences in the post hoc Dunn test (*a*: *p* < 0.001, *b*: *p*=0.017, *c*: *p*=0.018, *d*: *p*=0.017, *e*: *p*=0.010, and *f*: *p* < 0.001).

**Table 4:** Age mean values, median values, standard deviations, and the range by a tumor type.

Age, years	*n*	Mean (SD)	Median (range)	^*∗*^ *p*
Cyst	37	43.94 ± 20.52	39.00 (20.00; 86.00)^***a***^	0.006
Solid iris tumor	26	63.80 ± 14.96	65.00 (23.00; 86.00)^***a***^	
Ciliary body tumor	7	64.60 ± 14.26	62.00 (47.00; 81.00)	
Corneal tumor	4	62.25 ± 16.15	59.00 (48.00; 83.00)	

^*∗*^Kruskal–Wallis test; *a*: significant difference in the post hoc Dunn test (*p*=0.002).

**Table 5:** Tumor type between females and males.

Tumor type	Females	Males	^*∗*^ *p*
Cyst	28 (45.2)	9 (33.3)	0.038
Solid iris tumor	26 (35.5)	7 (25.9)	
Ciliary body tumor	3 (4.8)	4 (14.8)	
Anterior synechiae	1 (1.6)	2 (7.4)	
Corneal tumor	3 (4.8)	1 (3.7)	
Other	1 (1.6)	4 (14.8)	

^*∗*^Fisher's exact test; data presented as *n* (% of sex).

**Table 6:** BCVA mean values, median values, standard deviations, and the range by a tumor type.

BCVA	*n*	Mean (SD)	Median (range)	^*∗*^ *p*
Cyst	37	0.87 ± 0.25	1.00 (0.20; 1.00)^***a***^	0.038
Solid iris tumor	33	0.82 ± 0.20	0.85 (0.50; 1.00)	
Others	11	0.58 ± 0.35	0.50 (0.05; 1.00)^***a***^	

^*∗*^Kruskal–Wallis test; *a*: significant difference in the post hoc Dunn test (*p*=0.016).

**Table 7:** IOP mean values, median values, standard deviations, and the range by a tumor type.

IOP, mmHg	*n*	Mean (SD)	Median (range)	^*∗*^ *p*
Cyst	37	15.71 ± 2.78	16.00 (10.00; 20.00)	0.747
Solid iris tumor	33	15.00 ± 2.89	14.00 (12.00; 20.00)	
Others	11	15.86 ± 3.48	17.00 (11.00; 21.00)	

^*∗*^Kruskal–Wallis test.

**Table 8:** Correlation between tumor size and age, BCVA, and IOP.

Correlation with tumor size	Base width		Height	
	Spearman's rank correlation coefficient *r*_*s*_	*p*	Spearman's rank correlation coefficient *r*_*s*_	*p*
Age, years	0.38	0.003	−0.11	0.390
BCVA	−0.42	0.014	−0.15	0.420
IOP	−0.31	0.113	−0.02	0.939

## Data Availability

The data used to support this study can be obtained from the corresponding author upon request. The names and personal data of the participants cannot be released due to ethical aspects.
